# Transcriptional Profiling of Adult Neural Stem-Like Cells from the Human Brain

**DOI:** 10.1371/journal.pone.0114739

**Published:** 2014-12-16

**Authors:** Cecilie Jonsgar Sandberg, Einar O. Vik-Mo, Jinan Behnan, Eirik Helseth, Iver A. Langmoen

**Affiliations:** 1 Vilhelm Magnus Lab, Institute for Surgical Research and Department of Neurosurgery, Oslo University Hospital, Oslo, Norway; 2 Cancer Stem Cell Innovation Center (CAST), Oslo University Hospital and University of Oslo, Oslo, Norway; 3 Norwegian Stem Cell Center, Oslo University Hospital and University of Oslo, Oslo, Norway; University of Oxford, United Kingdom

## Abstract

There is a great potential for the development of new cell replacement strategies based on adult human neural stem-like cells. However, little is known about the hierarchy of cells and the unique molecular properties of stem- and progenitor cells of the nervous system. Stem cells from the adult human brain can be propagated and expanded *in vitro* as free floating neurospheres that are capable of self-renewal and differentiation into all three cell types of the central nervous system. Here we report the first global gene expression study of adult human neural stem-like cells originating from five human subventricular zone biopsies (mean age 42, range 33–60). Compared to adult human brain tissue, we identified 1,189 genes that were significantly up- and down-regulated in adult human neural stem-like cells (1% false discovery rate). We found that adult human neural stem-like cells express stem cell markers and have reduced levels of markers that are typical of the mature cells in the nervous system. We report that the genes being highly expressed in adult human neural stem-like cells are associated with developmental processes and the extracellular region of the cell. The calcium signaling pathway and neuroactive ligand-receptor interactions are enriched among the most differentially regulated genes between adult human neural stem-like cells and adult human brain tissue. We confirmed the expression of 10 of the most up-regulated genes in adult human neural stem-like cells in an additional sample set that included adult human neural stem-like cells (n = 6), foetal human neural stem cells (n = 1) and human brain tissues (n = 12). The NGFR, SLITRK6 and KCNS3 receptors were further investigated by immunofluorescence and shown to be heterogeneously expressed in spheres. These receptors could potentially serve as new markers for the identification and characterisation of neural stem- and progenitor cells or as targets for manipulation of cellular fate.

## Introduction

The discovery of adult neurogenesis and adult human neural stem-like cells (ahNSC) in the brain has opened a novel field of research aiming to utilise these cells as sources of repair in the treatment of degenerative disorders, such as Parkinson's and Alzheimer's disease [1]. ahNSCs can be isolated from the hippocampus or subventricular zone of the lateral ventricles (SVZ) [2]–[6]. Like stem cells from the rodent brain [7] they can be propagated and expanded *in vitro* as free floating neurospheres that are capable of self-renewal and can differentiate into all the three principal cell types of the central nervous system; neurons, oligodendrocytes, and astrocytes [6]. We have earlier shown that this includes neurons that generate action potentials [2], [3] and communicate by synapses [2].

Despite the great interest in and potential of ahNSCs, there is still limited knowledge regarding the hierarchy of stem- and progenitor cells in the human brain. This is in contrast to research on the hematopoietic cell lineage, where a detailed set of surface markers and transcription factors have been identified [8], [9]. Since the first successful attempt to phenotypically purify hematopoietic stem- and progenitor cells by simply depleting the lineage-restricted cells, the gradual discovery of new markers has made it possible to discriminate between long-term stem cells and more restricted progenitor populations. Similar approaches have been used in attempts to identify markers that prospectively distinguish adult NSCs from neural progenitors in rodents. Most markers are however common to several stages [10]. Studies of NSCs from the adult human brain are still few and far between, although GFAPδ positive cells expressing the surface receptor NGFR was recently suggested as a markers of ahNSCs [11].

The exploration of the ahNSC lineage is undoubtedly challenged by our limited access to human brain tissues. To our knowledge, only two reports have directly explored the global transcriptome of adult human stem- and progenitor cells cultivated as free floating spheres. The cells explored in these studies were derived from the hippocampus and olfactory bulb and included only cells from one and two patients, respectively [12], [13]. Additional investigations of adult stem- and progenitor cells from other parts of the human brain, not least the subventricular zone, is therefore necessary. Furthermore, selective markers that allow for a better separation of the different cell types cells in the lineage must be identified, and more efficient growth conditions to generate a sufficient number of cells both for research and patient treatment must be developed.

We have recently compared ahNSCs to glioma stem cells (GSCs) and identified dysregulated pathways and putative targets for the treatment of glioblastoma [14]. In this report, we further explore the transcriptome of ahNSCs, comparing it to normal adult cells including tissues from the brain, foetal brain and spinal cord. We define a gene expression pattern that is specific for human SVZ-derived ahNSCs and validate these findings in an additional sample set using quantitative PCR (qPCR) analysis and immunoflourescence.

## Materials and Methods

### Cell Culture

The tissue harvesting was approved by the Norwegian National Committee for Medical Research Ethics (07321b) and participants provided written informed consents. The biopsies were placed in ice cold Leibowitz-15 medium (L15, Invitrogen, Carlsbad, CA, USA). A biopsy was washed in L-15 and mechanically dissociated using two scalpels. The dissociation of the samples into single cells was achieved by incubation in papain (13,2 U/ml, Worthington Biochemical Corporation, Lakewood, New Jersey, USA) for 2×5 minutes at 37°C and gentle tituration with a pipette. After dissociation, papain activity was blocked using 2 mg/ml human albumin (Octapharma Pharmazeutika Produktionges, Wien, Austria), and the cells were washed in L-15 twice, and transferred to proliferation medium containing 10 ng/ml basic fibroblast growth factor (bFGF), 20 ng/ml epidermal growth factor (EGF) (both obtained from R&D Systems, Minneapolis, MN, USA), 10 ng/ml Leukemia inhibitory factor (Millipore, Billerica, MA, USA), B27-supplement 1∶50 (Invitrogen), 100 U/ml of both penicillin and streptomycin (Lonza, Basel, Switzerland), heparin 1 ng/ml (Leo Pharma, Ballerup, Denmark), and 8 mM HEPES (Lonza) in Dulbecco's modified essential medium with nutrient mix F-12 and Glutamax (DMEM/F12) (Invitrogen). The cells where then incubated at 10^5^ cells/ml in a 75 cm^2^ non-treated cell culturing flask (Nunc, Roskilde, Denmark). The cells in culture proliferated as free-floating spheres. When the spheres reached a size where the core of the spheres turned dark (70–100 µm, 22±8 days), cultures were enzymatically digested to single cells. The cells where then replated at 5×10^4^ cells/ml. The secondary spheres from each primary culture were harvested for RNA isolation or immunofluorescence.

The foetal human neural stem cells used for qPCR analysis were from the v-myc-immortalized ReNcell VM cell line (Chemicon, Temecula, CA, USA). They were cultivated as neuorspheres as described above and harvested at passage two.

### Cell viability

After digestion of spheres to single cells, the cell number and viability was determined using the Nucleocounter system according to the manufacturers' instructions (Nucleocounter, Chemometec, Allerod, Denmark). The method allows detection of non-viable and viable cells in cell suspensions using propidium iodide (PI). The sphere viability was also investigated in intact spheres with fluorescein diacetate (FDA) and PI. Spheres were spun down by centrifugation and incubated for 3 minutes in 0.02 mg/ml FDA and 0.1mg/ml PI at room-temperature. Labeled cells were imaged using an Olympus IX81 inverted fluorescence microscope. Images were acquired using Olympus soft imaging Excellence software. Post processing of the images was done using the ImageJ software (http://imagej.nih.gov.com).

### RNA isolation

Total RNA from spheres and tissue biopsies was isolated using Qiazol and the RNeasy Micro Kit (Qiagen GmbH, Hilden, Germany). The concentration of each RNA sample was determined by using a Nanodrop spectrophotometer, and the samples were analyzed for quality using an Agilent 2100 Bioanalyzer with the RNA Nano Assay. Only RNA samples with a RIN value greater than eight were included in further analyses. Total RNA from foetal human brain was purchased from Clontech (Clontech Laboratories, CA, USA).

#### Microarray hybridisation and analysis of microarray data

RNA from each sample was reverse transcribed and amplified using the NanoAmp RT-IVT Labelling Kit (Applied Biosystems). The DIG-labelled cRNA (10 µg) was fragmented and hybridised to Applied Biosystems Human Genome Survey Microarray V2.0 (Applied Biosystems) slides according to the manufacturer's protocol (Applied Biosystems Chemiluminescence Detection Kit). The slides were scanned using an ABI 1700 Chemiluminescent Microarray Analyzer. An initial analysis was performed using J-Express 11 software (Molmine). After removing the flags and control spots, the arrays were quantile normalised and log2-transformed. Further analyses and statistics were performed using J-Express (Molmine) and WebGestalt [15] software. Differential gene expression analysis was carried out using the rank product method [16]. Pathways and gene ontology were obtained from KEGG [17] and Gene Ontology [18]. The microarray data have been submitted to the Gene Expression Omnibus (GSE31262).

#### Quantitative real-time PCR

Quantitative PCR was performed according to the manufacturer's instructions (High Capacity cDNA Reverse Transcription Kit) using TaqMan Universal PCR Master Mix and the ABI Prism 7900 Sequence Detection System with the associated software (Applied Biosystems, Foster City, CA). The following oligonucleotide primers and probes were purchased from TaqMan Applied Biosystems: Hs00159616_m1(*NKX2-2*), Hs00536106_s1(*SLITRK6*), Hs00226053_m1(*RNF128*), Hs00193613_m1(*PLXNB3*), Hs01931464_s1(*TMEM37*), Hs00171558_m1 (*TIMP1*), Hs00230757_m1(*EMILIN2*), Hs00182120_m1(*NGFR*), Hs00158478_m1(*KCNS3*), Hs00167155_m1(*SERPINE1*), Hs99999903_m1(*ACTB*), Hs00707120_s1 (*NES*), and Hs01053049_s1(*SOX2*). Human *ACBT* (TaqMan endogenous control reagents, Applied Biosystems) was used as a housekeeping gene. Standard curves were obtained from serial dilutions of total RNA. The reverse transcription and qPCR of each sample were run in triplicate. The thermal cycling conditions were as follows: 2 min at 50°C and 10 min at 94.5°C, followed by 40 cycles of 30 s at 97°C and 1 min at 59.7°C. The relative gene expression levels were calculated using the standard curve method (Manual: Relative Quantitation of Gene Expression: ABI PRISM 7700 Sequence Detection System: User Bulletin #2: Rev B). Differences in gene expression were assessed by independent sample t-test.

### Immunofluorescence

Spheres and tissue biopsies were fixed in 4% paraformaldehyde in phosphate buffered saline (PBS) pH8, cryoprotected in 20% sucrose and incubated in OCT (Tissue-TEK, Sakura Finetek, CA, USA). The blocks were then cryosectioned at 10 or 20 µm on a freezing microtome and thawed onto Super Frost/Plus microscope slides (Menzel-Gläzer, Braunschweig, Germany). Alternatively, the spheres were treated with papain to generate single cells (as described under cell culture), seeded on glass-bottom dishes coated with fibronectin (Sigma Aldrich), and incubated over night before being immunolabelled. For immunolabelling, the sections were first rehydrated in PBS, permeabilized (0.1% Triton in PBS) and blocked for one hour at room temperature (5% bovine serum albumin (BSA) and 5% donkey serum in PBS). The sections were further incubated in the indicated primary antibody overnight at 4°C. Rabbit anti-NGFR (1∶1000, Cell Signaling Technology), rabbit anti-KCNS3 (1∶100, Sigma Aldrich), rabbit anti-SLITRK6 (1∶25, Merck Millipore) and mouse anti-SOX2 (1∶200, Cell Signaling Technology) antibodies were used in this study. The sections were then washed and incubated with secondary antibodies for one hour at 4° C (anti-rabbit Alexa 488 and anti-mouse Alexa 488, both 1∶1000, Molecular Probes). Nuclear staining was performed using Hoechst 33358 (1∶5000, Sigma-Aldrich). Analysis and image acquisition was done on an Olympus BV 61 FluoView confocal microscope (Olympus, Hamburg, Germany), using FV10-ASW 1.7 software (Olympus).

## Results

### Characterisation of the neurosphere cultures

**Figure 1:**
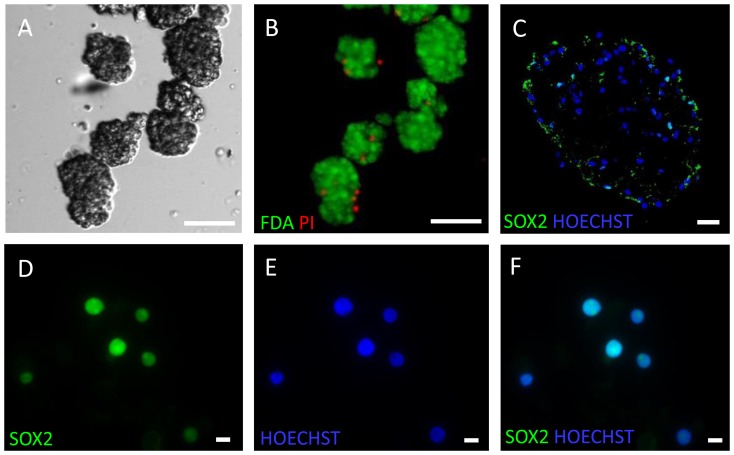
ahNSCs cultivated as neurospheres display high viability and express SOX2. (**A**) The neurospheres were collected when their size reached a diameter of 70–100 µM. Scalebar 100 µM (**B**) The fraction of viable cells in neurospheres (green, FDA) was high compared to the fraction of dead cells (red, PI) (**C**) Neurosphere with SOX2 positive cells (green) and Hoechst stained nuclei (blue). Scalebar 10 µM (**D**) Single cells from neurosphere cultures with SOX2 positive nuclei (green). (**E**) Nuclear staining of single cells with Hoechst (blue) (**F**) Merge of E and F.

Neurospheres with an average diameter of 70-100 µm were formed after 22±8 days. The cell viability was generally high, tested both in single cell suspensions immediately after enzymatic dissociation (85±15, n = 6) and in intact spheres with fluorescent probes for live-dead staining with fluorecent probes (FDA/PI) (**Fig. 1 A–B** **). We and others have earlier characterised the structure and expression of markers such as nestin and SOX2 in human-derived neurospheres from the SVZ [4], [19]–[21]. As a positive control in this study, SOX2 expression was confirmed both in intact, cryosectioned spheres and in single cells from dissociated neurospheres (Fig. 1 C–F**).

### Unsupervised hierarchical clustering of ahNSCs with human tissues

**Figure 2:**
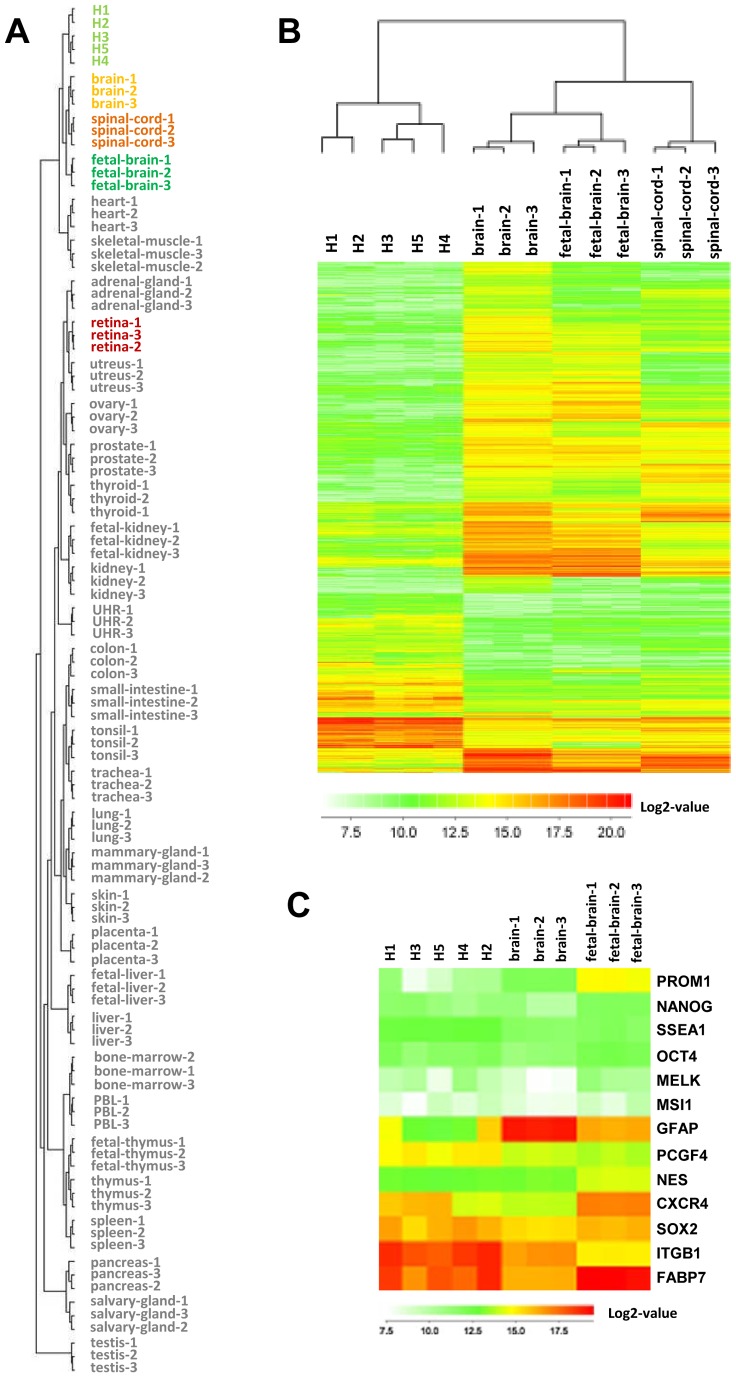
Identification of a specific ahNSCs gene expression pattern. (**A**) Unsupervised hiererarchical clustering analysis based on the global gene expression of five samples of ahNSCs (green) and 31 normal human tissue samples (in triplicate). ahNSCs clustered together with brain-, spinal cord- and tissue foetal brain tissue (**B**) Unsupervised hierarchical clustering analysis based on gene expression of selected genes (rank product analysis, 1% FDR) in five ahNSCs samples and tissue samples (in triplicate) from brain, foetal brain, and spinal cord. The ahNSCs clustered separately from the tissues. (**C**) Heat-map illustrating the expression of typical stem cell markers in the ahNSCs, brain tissue and foetal brain tissue.

Gene expression data from five individual samples of ahNSCs were analysed together with an external dataset from the same microarray platform (accession number: GSE7905). This dataset included the gene expression data from thirty-one normal human tissues, including samples from the nervous system (brain, foetal brain, and spinal cord). All of the samples were initially quantile normalised using J-Express (Molmine). Unsupervised hierarchical clustering analysis based on the overall gene expression profiles of these samples indicated that ahNSCs clustered together with brain, spinal cord- and foetal brain tissue (**Fig. 2A**). An analysis of the transcriptome dynamics between ahNSCs and brain tissue revealed that 1,189 transcripts were significantly modulated (rank product algorithm, 1% false discovery rate (FDR)) (S1 **Table**). Unsupervised hierarchical clustering analysis of these 1,189 genes in a smaller set of samples including ahNSCs and neural tissues separated the samples in two main groups: one cluster with ahNSCs and one cluster with all neural lineage tissues (**Fig. 2B**). Of the genes that were differentially expressed between ahNSCs and adult brain tissue, only 30% (351 genes) were up-regulated in ahNSCs.

### Genes and pathways of significance

We have previously examined neurospheres using immunofluorescence and found that they express typical stem- and lineage markers such as NES, GFAP and SOX2 [14], [19]. The most highly expressed markers in the microarrays of ahNSCs were *FABP7*, *ITGB1*, *SOX2* and *CXCR4* (**Fig. 2C**). The polycomb group gene *PCGF4* (also known as *BMI1*) was expressed at a higher level in ahNSCs than in brain tissue. *PROM1* (also known as *CD133*) was highly upregulated in foetal brain tissue. Some markers that are typically expressed in differentiated cells, such as *GFAP* and *MAP2* were downregulated in ahNSCs compared to brain tissue, whereas the oligodendrocyte marker *OLIG2* was more highly expressed in the ahNSCs.

A pathway analysis including both up- and downregulated genes revealed that the most significantly altered pathways were calcium signalling, neuroactive ligand-receptor interactions and MAPK signalling (**Table 1** **and** **S1 Table**). However, all of the pathway categories were dominated by genes that were downregulated in ahNSCs; only a few of the pathway-annotated genes were upregulated in ahNSCs. Among these were signalling ligands (*FGF19*, *WNT1*), several axon guidance receptors (*SEMA3C*, *UNC5*, *ROBO1*, and *PLXNB3*), and *TP53*.

**Table 1:** Results from pathway analysis of genes regulated in ahNSCs (Rank 1%).

KEGG pathway	No of genes regulated (<> 5-fold) up/down	p-value enrichment
Calcium signaling pathway	2/21	4.9e-14
Neuroactive ligand-receptor interaction	2/24	1.77e-13
MAPK signaling pathway	4/21	1.02e-10
Long-term potentiation	0/12	1.04e-08
Axon guidance	3/13	1.32e-08
Vascular smooth muscle contraction	1/11	1.04e-07
Dilated cardiomyopathy	1/11	2.46e-07
Amyotropic lateral sclerosis (ALS)	1/10	2.69e-07
Melanogenesis	1/9	7.39e-07
Regulation of actin cytoskeleton	4/13	9.71e-07

**Figure 3:**
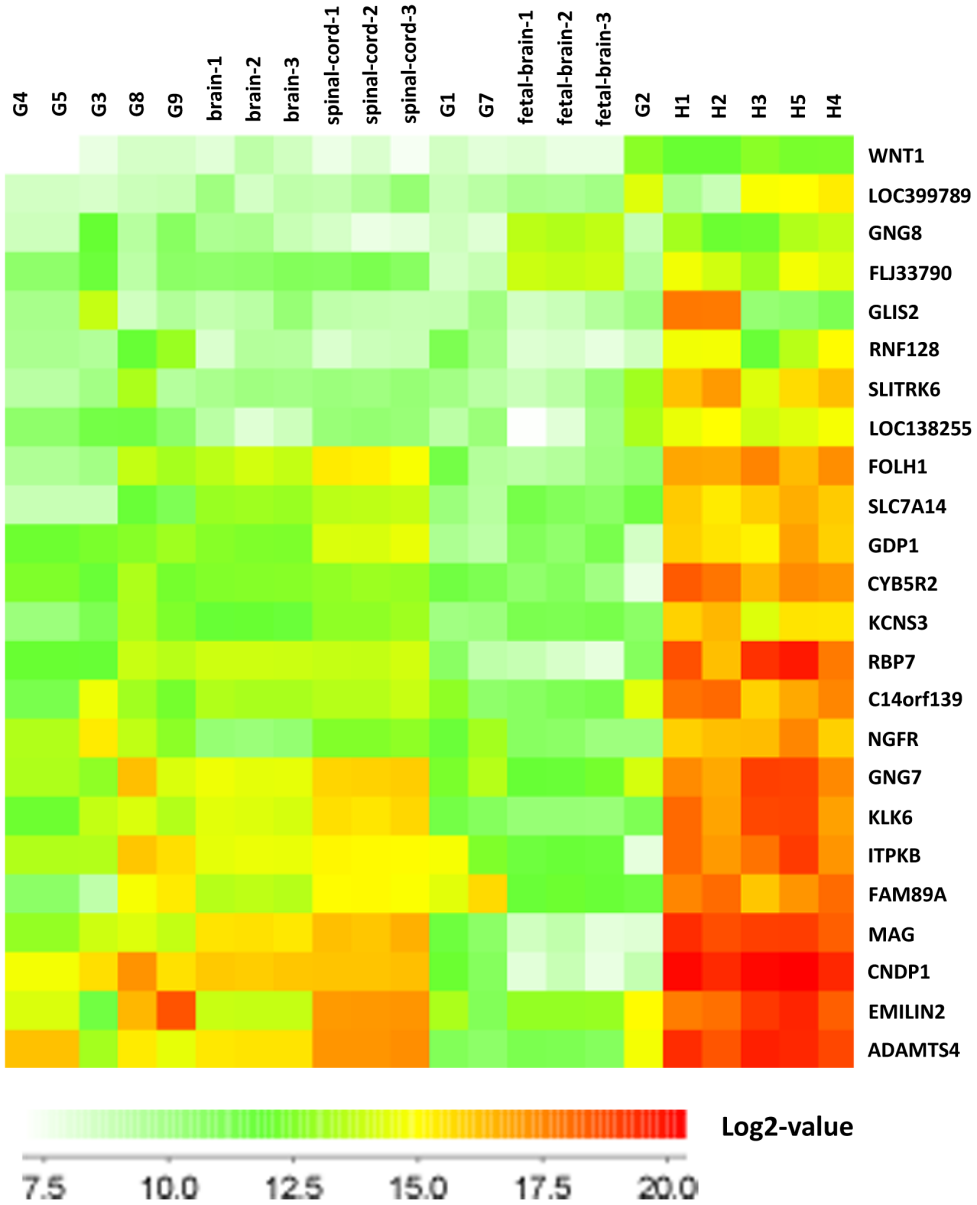
Heat-map of 24 genes that are highly overexpressed in ahNSCs compared to normal brain tissue. The expression of GSCs, spinal cord and foetal brain tissues are also presented.

We further categorised the most highly upregulated genes in ahNSCs according to gene ontology categories (rank product algorithm, 0.1% FDR, 154 genes) (S1 **Table**). We observed that many of these genes are associated with biological processes during development and encode proteins localized in the extracellular region of cells (**Table 2**, S1 **Table**). Several of the ahNSC-upregulated genes encode transcription factors (*NKX6-2*, *NKX2-2*, *PBX3*, *GLIS2*, and *ZNF161*) and proteases/protease-inhibitors (*MMP19*, *ADAM9*, *KLK6*, *ADAMTS4*, *TIMP1*, *TIMP2*, and *SERPINE1*). Twenty four out of the 154 genes were also highly expressed in ahNSCs compared to GSCs (cultured under identical conditions as ahNSCs) (**Fig. 3**). This subset included several of the aforementioned proteases as well as metabolism-related genes (*CYB5R2*, *GPD1* and *ITPKB*). *WNT1*, a ligand in the Wnt-pathway that is known to play a role in CNS-development, was 12.5-fold upregulated. Most interestingly, we identified five plasma-membrane receptors (*NGFR*, *SLITRK6*, *KCNS3*, *TMEM37* and *PLXNB3*).

**Table 2:** Results from gene onthology analysis of genes up-regulated in ahNSCs (Rank 0.1%, 154 genes).

Onthology	No of genes regulated (<> 5-fold)	p-value enrichment
**Biological process**		
Anatomical structure development	62	9.75e-05
Developmental process	71	9.75e-05
System development	58	0.0001
Organ development	44	0.0002
Multicellular organismal development	63	0.0002
Membrane protein proteolysis	25	0.0004
Anatomical structure morphogenesis	34	0.0021
Membrane protein ectodomain proteolysis	5	0.0021
Tissue development	24	0.0021
Cellular developmental process	37	0.0037
**Cellular component**		
Extracellular region	48	0.0011
Extracellular region part	29	0.0011
Extracellular matrix	14	0.0049

### Verification of identified genes in an additional set of samples

**Figure 4:**
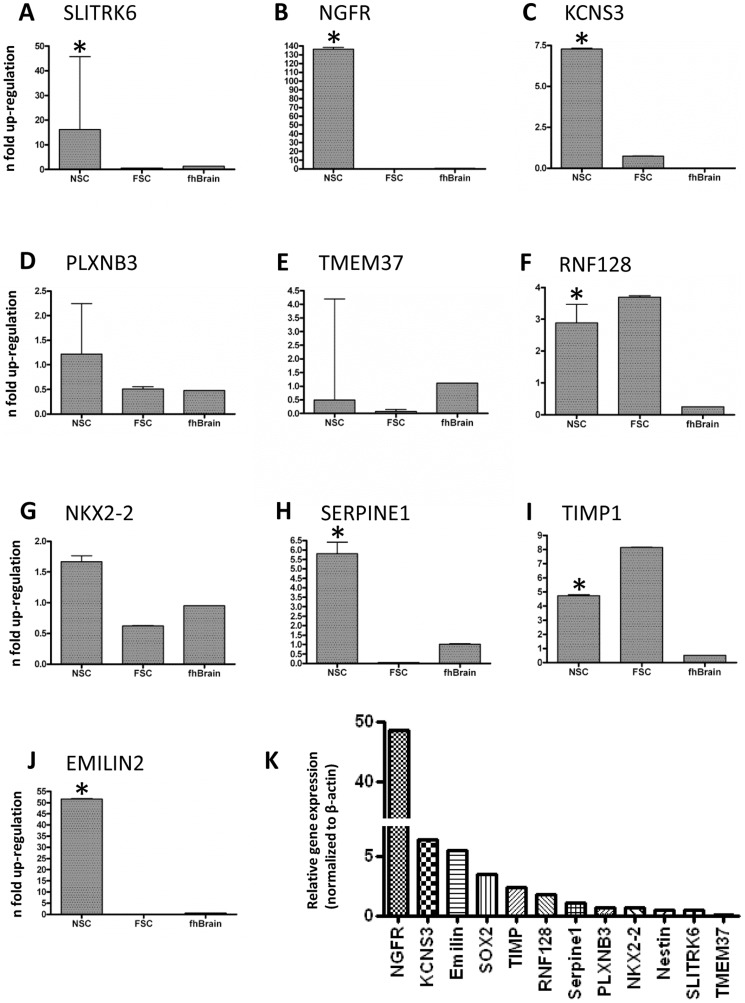
Relative gene expression of selected genes in ahNSCs. (**A–J**) qPCR analysis of ahNSCs (n = 6), fhNSCs (n = 1, 3 replicates), foetal human brain tissue (n = 1, 3 replicates) normalized to adult human cortex tissue (n = 12). The error bars represent the standard deviations. NSC-bars with * indicate significantly increased gene expression level (p<0.005) compared to adult human cortex tissue. (**K**) Relative gene expression levels normalised to *ACTB*.

To examine whether our findings from the microarray analysis are relevant in a broader set of samples, we collected a new dataset including six samples of ahNSCs and 12 samples of human cerebral cortex. For comparison, we additionally included a commercial foetal neural stem cell line (fhNSCs), which was grown under the same conditions as the ahNSCs, and an RNA sample from foetal human brain tissue (fHB) (Clontech). We analysed *NES* and *SOX2* as well as 10 selected genes that were highly upregulated in ahNSCs by qPCR (**Fig. 4**).

Of the five membrane receptors investigated (Fig. 4**A–E**); we could confirm that *NGFR* (Fig. 4**B**) and *KCNS3* (Fig. 4**C**) were highly and selectively expressed in ahNSCs. *NGFR* was not present in either the brain tissue, fHB, or FSCs. *KCNS3* was only expressed in the FSCs and ahNSCs. We also observed that *SLITRK6* was highly expressed in ahNSCs but that its expression level varied significantly between individuals (Fig. 4**A**). Several proteases were enriched in ahNSCs; of these, *SERPINE1* and *TIMP1* were investigated and confirmed (Fig. 4**H, I**). Moreover, we confirmed the upregulation of two transcription factors that are known to be important in development: *RNF128* and *NKX2-2* (Fig. 4**F, G**). *RNF128* was also highly expressed in the FSCs. *EMILIN2*, which encodes an extracellular matrix protein, was almost exclusively expressed in the ahNSCs (Fig. 4**J**). Among the genes examined, *NGFR* was expressed at a far higher level than the others (Fig. 4**K**).

### SLITRK6, NGFR and KCNS3 are highly expressed in ahNSCs

**Figure 5:**
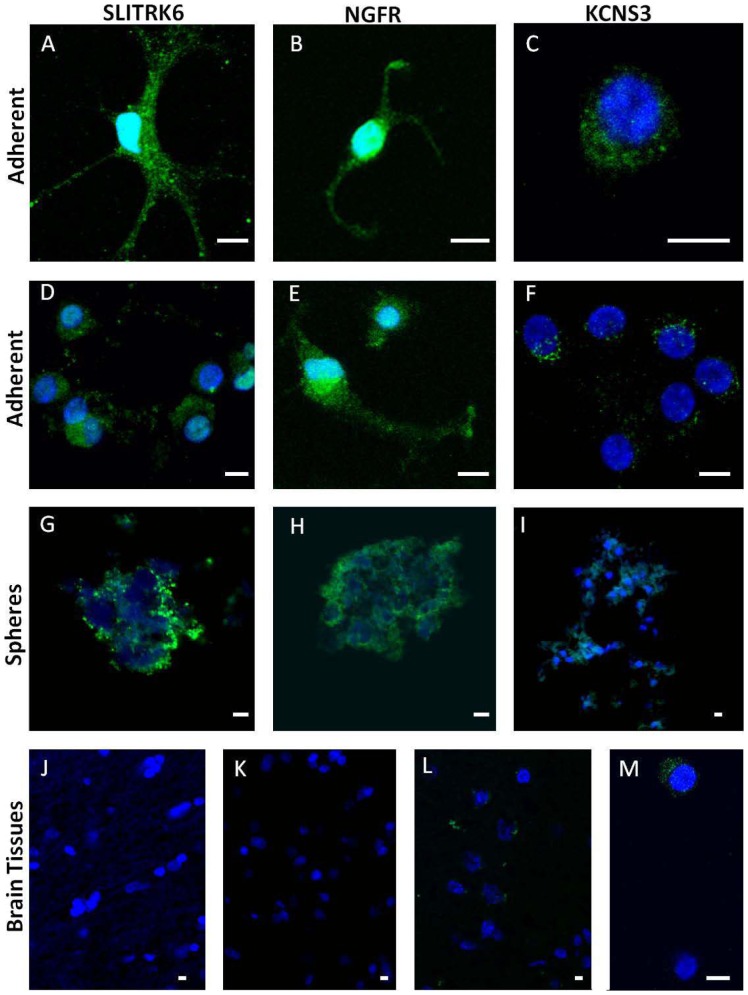
Immunofluoresence of selected plasma-membrane receptors. SLITRK6 (**A–D–G**), NGFR (**B–E–H**) and KCNS3 (**C–F–I–M**) in ahNSCs. The cells were examined as either single cells adhering to fibronectin (adherent) (**A–F, M**) or as intact, sliced spheres (spheres) (**G–I**). The sections from human cerebral cortex were negative for SLITRK6 (**J**), NGFR (**K**) and KCNS3 (**L**). All scalebars are 10 µM.

The identification and characterisation of ahNSCs is hampered by the lack of phenotypic markers. Plasma membrane receptors are particularly useful as immunotargets for cell-sorting experiments and the exploration of subgroups in a heterogeneous cell population. With this consideration in mind, we used immunofluorescence to further examine the expression of SLITRK6, NGFR and KCNS3 (**Fig. 5**). The spheres were either directly fixed or enzymatically digested and adhered to fibronectin-covered glass dishes overnight before fixation. All three receptors were expressed, but SLITRK6 (Fig. 5**A, D, and G**) and NGFR (Fig. 5**B, E, and H**) exhibited a stronger staining than KCNS3 (Fig. 5**C, F, I and M**). The receptors were heterogeneously expressed (Fig. 5**D, F, and M**) and were not expressed in human brain cortex tissue (Fig. 5**J, K, and L**).

## Discussion

To our knowledge, this study represents the first global transcriptional profile of ahNSCs from the subventricular zone enriched as neurospheres. From 12 individuals, including samples for microarrays, qPCR and immunofluorescence, we present the genes and pathways that are highly upregulated in ahNSCs compared to levels in normal brain tissue. Our cells were cultured under sphere-forming conditions *in vitr*o; this approach is by far the best-characterized method to enrich for both neural stem cells and many other types of somatic stem cells. The method has been shown to be a robust method for enrichment of stem-like cells from a range of species (murine [22], canine [23], porcine [24], monkey [25] and human [4]), organs and malignancies (colon [26], [27], breast [28], [29], prostate [30], heart [31], skin [32], brain [33]–[37] pancreas and liver [38]). ahNSCs that are enriched as neurospheres are most likely a heterogeneous mixture of stem- and progenitors [21]. No well-defined molecular identity of each cell type has been established, and the hierarchy speculated to be present in these spheres has not been confirmed. Several stem cell markers, such as nestin and SOX2, have been identified and shown to label neural stem cells in the SVZ both *in vitro* and *in vivo.* However, these markers are not useful in discriminating between the different cell types alone, because they are also expressed by more restricted progenitors [39]–[41]. The genes identified in our study may serve as new and useful identifiers for further exploration of the heterogeneity of neurospheres and may lead to a better discrimination between stem cells and progenitor cells.

The chemokine receptor CXCR4 was highly upregulated in ahNSCs. This is in line with previous findings from our lab. We have earlier identified high fractions of CXCR4-positive cells in SVZ-derived neurospheres [19], and ahNSCs transplanted to an ischemic rat model expressed CXCR4 [42]. Signaling through the SDF-1/CXCR4 pathway has interestingly been shown to be an important intercrine member in promoting the directed migration of stem cells in CNS injury [43] and regulate both the migration and proliferation of adult neural stem-like cells in rodents [44], [45]. Furthermore, CXCR4 plays a role in HIV were the receptor is used by several of the HIV proteins to enter cells in the CNS. This may lead to inflammation and cell death [46].

The only other studies that are directly comparable to ours explored ahNSCs derived from the hippocampus [12] or olfactory bulb tissue [13] and did only include one and two patient samples, respectively. Unfortunately, the original data from these studies are not publically available, and we were not able to statistically compare those results to our data. However, the lists of genes enriched from the previous two studies did have several genes in common with the list of genes that we identified as highly expressed in ahNSCs from the SVZ. Of the 45 genes being presented as highly enriched in spheres derived from the hippocampus, 39 were also highly expressed in SVZ-derived ahNSCs. Twenty of these genes were also significantly upregulated in SVZ-derived ahNSCs (within 5% FDR), including *NGFR*, *SERPINE1*, *TIMP1*, *OLIG2*, *ADAM9* and *FABP5*. We also identified similarities between the patterns of gene expression in SVZ- and olfactory bulb-derived NSCs. Of the 84 genes that were highlighted as highly expressed in the olfactory bulb-derived ahNSCs, 43 were also highly expressed in SVZ-derived ahNSCs. Only six of these were significantly upregulated in our dataset (within 5% FDR), but interestingly these six shared genes included *NGFR*, *OLIG2*, *WNT1*, *ADAM9*, *KLK6* and *SLCA15*. These findings support that the three sources of ahNSCs have molecular programs in common even though derived from totally different regions of the brain [47].

fhNSCs have been explored in various ways, including with global gene expression analysis, because they are an alternative source of cells for therapy [48], [49]. Maisel et al. compared their hippocampus-derived ahNSCs to fhNSCs and found that although there were similarities in gene expression, the two cell types utilised divergent paths to maintain their cellular state [12]. Our analysis directly comparing gene expression in ahNSCs and fhNSCs is in line with this result; our quantitative PCR analysis of selected genes identified significant differences between the cell types.

*NGFR* was one of the most upregulated and highly expressed receptors in both our study using SVZ-derived cells and the two comparable studies using adult derived NSCs [12], [13]. NGFR is widely expressed in the nervous system during development, but its expression decreases dramatically by adulthood [50]. NGFR has been shown to be absent in a wide range of adult CNS tissues [51]. It can be re-expressed in the nervous system in states related to neural cell death or enhanced survival of neurons and glia when co-expressed with TrkA [50]. NGFR was recently suggested to be a marker for GFAPδ-positive ahNSCs [11]. High-level expression of NGFR has also been suggested as a marker for neural crest stem cells [52], oligodendrocytic precursor cells [53] and melanoma cancer stem cells [54].

SLITRK6 is one of six members in the Slitrk family of transmembrane proteins that shares significant homology with the neurite-modulating proteins Slit and Trk. Although SLITRK6 was significanly upregulated ahNSC, its expression varied higly between the samples compared to the minimal variation identified in NGFR expression. This indicates that the SLITRK6 expression in ahNSCs varies from patient to patient, as we generally detect less than 10% differences in gene expression within samples analyzed from the same patient. The involvement of Slitrk proteins in neurite modulation has been verified in mouse and rat cell-lines [55]. With the exception of *SLITRK6*, the family of *SLITRK* genes is expressed in a broad range of neural tissues including the adult human brain. The *SLITRK6* is very different in that it is not expressed in the cerebral cortex and is mainly detected in the putamen of the adult human brain [56]. A role for *SLITRK* genes in undifferentiated cells has also been suggested because the genes are expressed by both hematopoietic and embryonic stem cells [57]. The finding of high SLITRK6 expression in neural stem and progenitor cells is novel.

KCNS3 (Kv9.3) is a subtype of 40 voltage gated potassium channels. It is not functional alone but co-assembles with Kv2 subunits and modifies their activity [58]. Its identification in ahNSCs is novel. Its role remains unclear.

In summary, we have used microarrays to identify the genes and pathways that are significantly up-regulated in ahNSCs compared to adult brain tissue. Using qPCR, we have confirmed the genes that are highly expressed in ahNSCs and either weakly or not present in the cortex of the adult human brain. Three of these genes were the receptors *NGFR*, *SLITRK6* and *KCNS3*; all were heterogeneously expressed in neurospheres. The identified genes and proteins should be further explored to understand their role in ahNSCs. NGFR, SLITRK6 and KSCNS3 may have potential as markers discriminating between different cell populations in the neural stem cell hierarchy and may be potential targets for cell manipulation.

## Supporting Information

S1 Table **Results from significance analysis of global gene expression in ahNSCs compared to tissue from the adult human brain.** The table includes results from Rank (1% FDR)-, pathway- and GO-analysis.
(XLSX)
